# Unilateral branch retinal artery occlusion in a patient with systemic lupus erythematosus: A case report

**DOI:** 10.1097/MD.0000000000029005

**Published:** 2022-03-11

**Authors:** Linglin Zhang, Chaoqiang Guan, Zuke Ye, Yan Lu

**Affiliations:** a Experimental and Translational Ophthalmology, Department of Ophthalmology, University Medical Center of the Johannes Gutenberg University, Langenbeckstr, Mainz, Germany; b Department of Ophthalmology, Foshan Second People's Hospital, 78 Weiguo Road, Chancheng District, Foshan, Guangdong Province, China.

**Keywords:** lupus, macula, retina, vaso-occlusion

## Abstract

**Rationale:**

Ocular involvements in systemic lupus erythematosus (SLE) are not rare but extremely varied. Here we present a very meaningful case with unilateral branch retinal artery occlusion (BRAO) secondary to undetected SLE, for which immunotherapy showed significant effect, which led to complete resolution of macular edema.

**Patient concerns:**

A 31-year-old female patient, so far without any previous illnesses, presented with a sudden onset of painless diminution of vision in left eye for 5 days. Diagnosis: Signs of branch retinal artery occlusion and macular ischemic edema were observed on the left fundus, which was further confirmed by Fundus fluorescein angiography and OCT. BRAO was initially proposed. However, after ophthalmological treatment for BRAO, visual acuity and macular edema improvement was limited. Physical examination revealed erythema on the hands and feet, together with her experience of hair loss and joint pain. The patient was diagnosed with SLE.

**Interventions and outcomes:**

The patient received systemic immunotherapy, which resulted in visual improvement to 20/20.

**Lessons:**

This is a rare monocular vaso-occlusive retinopathy in SLE with mainly diffuse nonperfusion and small arterial and arteriolar occlusion in the retina, as distinct from more common vasculitis. Even without intravitreal injection of anti-VEGF, systemic immunotherapy can be effective for the treatment of macular edema.

## 1 Introduction

Systemic lupus erythematosus (SLE) is a multisystem autoimmune disease with a chronic course and variable manifestation. SLE affects roughly 20 to 150 people per 100,000,^[1]^ with a higher incidence in women (a female/male ratio ranging from 6:1 to 10:1).^[2]^ The etiology of SLE is still undefined while the clinical onset derives from various factors including genetic predisposition, environmental stimuli, immunological and hormonal dysregulation.^[3]^ The presence the multiple autoantibodies, which led to formation and deposition of immune complexes (ICs), could be considered a disease hallmark.^[4]^ SLE can virtually affect any organ system, with a wide heterogeneity in terms of clinical manifestations requiring personalized treatments.^[5]^

SLE-related eye involvement could occur at any stage of the disease and is usually indicative of disease activity, which mostly involve both eyes. Keratoconjunctivitis sicca is the most frequent ophthalmic manifestation^[6]^ while retinal and choroidal involvement are most associated with visual loss.^[7]^ Retinopathy in SLE most commonly presents with microangiopathy manifesting as cotton-wool spots and intra-retinal hemorrhages.^[8]^ Vaso-occlusive retinopathy such as retinal arterial and venous occlusions are rarely described as initial manifestation in SLE patients. Symptomatic retinal vasculopathy has been reported in 0.66% of patients with SLE, which often leads to poor visual outcome, with more than one third of the involved eyes did not recover from bad vision.^[9]^ Here we reported a case of rare unilateral vaso-occlusive retinopathy in SLE, which characterized by extensive occlusion of branch retinal arteries and arterioles around the macula.

## 2 Case presentation

A 31-year-old woman visited our eye clinic complaining of vision loss in left eye. The patient had a sudden loss of vision in the left eye 5 days ago without any obvious inducement. There was no ocular redness, discharge, pain or periorbital swelling. She was married with twice normal obstetric experience. She had no previous history of being diagnosed with any disease.

**Figure 1:**
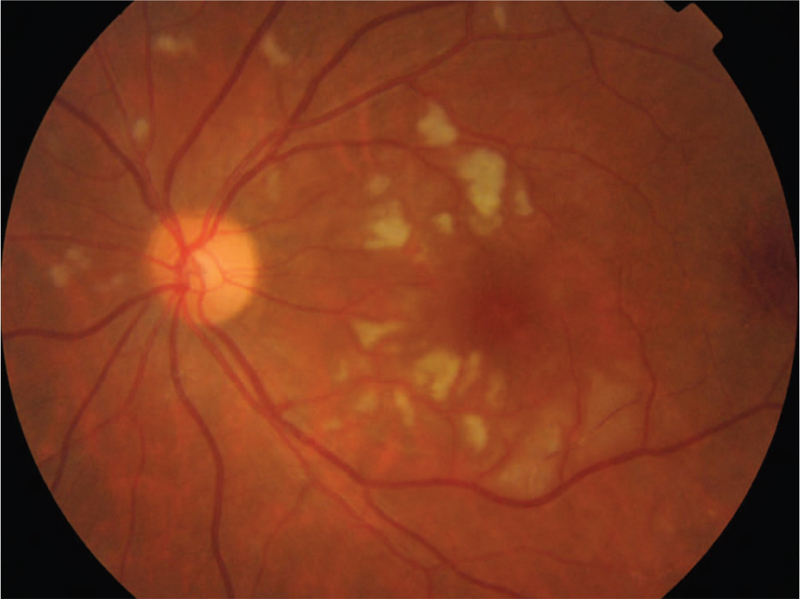
Fundus image of the left eye. There were several cotton-wool spots near the nasal and upper branch artery. There are gray-white areas of ischemic edema around the macula, between the branches of small arteries. Arteriolar attenuation was present near macula, middle-distal part of which was white thread-like occlusion. The branch retinal veins were slightly tortuous and dilated. In mid-peripheral and peripheral retina, blot hemorrhages and white-line like occluded arterioles can be clearly seen.

**Figure 2:**
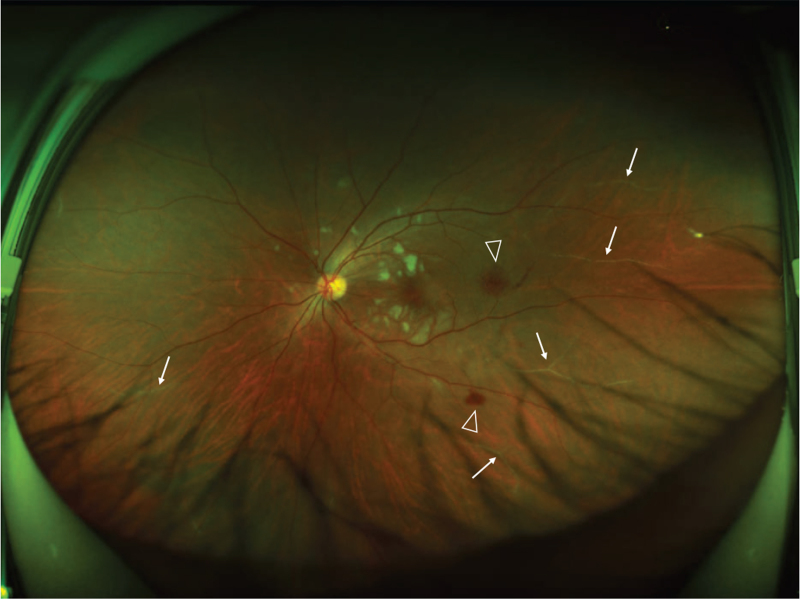
Scanning laser fundus image of the left eye. The middle section of the artery-the distal end is slender, white thread-like occlusion, the local small blood vessels under the nose and above the peripheral white sheath (indicated by the white arrow), and retinal hemorrhage (indicated by the triangle).

**Figure 3:**
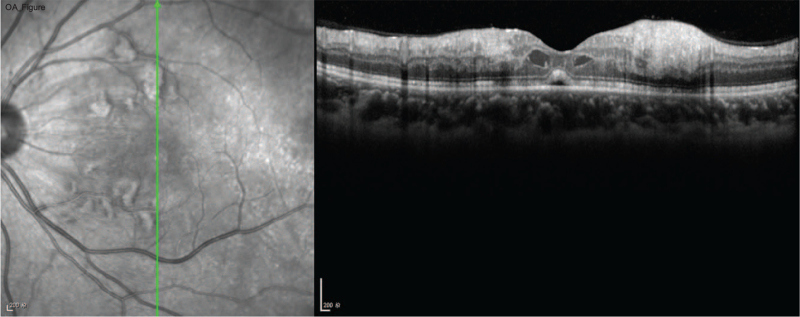
SD-OCT image of the left eye. A small focal bulge can be seen on the RPE layer of the retina in the macular area of the left eye. Macula is edema, with high reflection signals in the inner layer, and cystic low reflection signals between the foveal nerve and epithelium.

**Figure 4:**
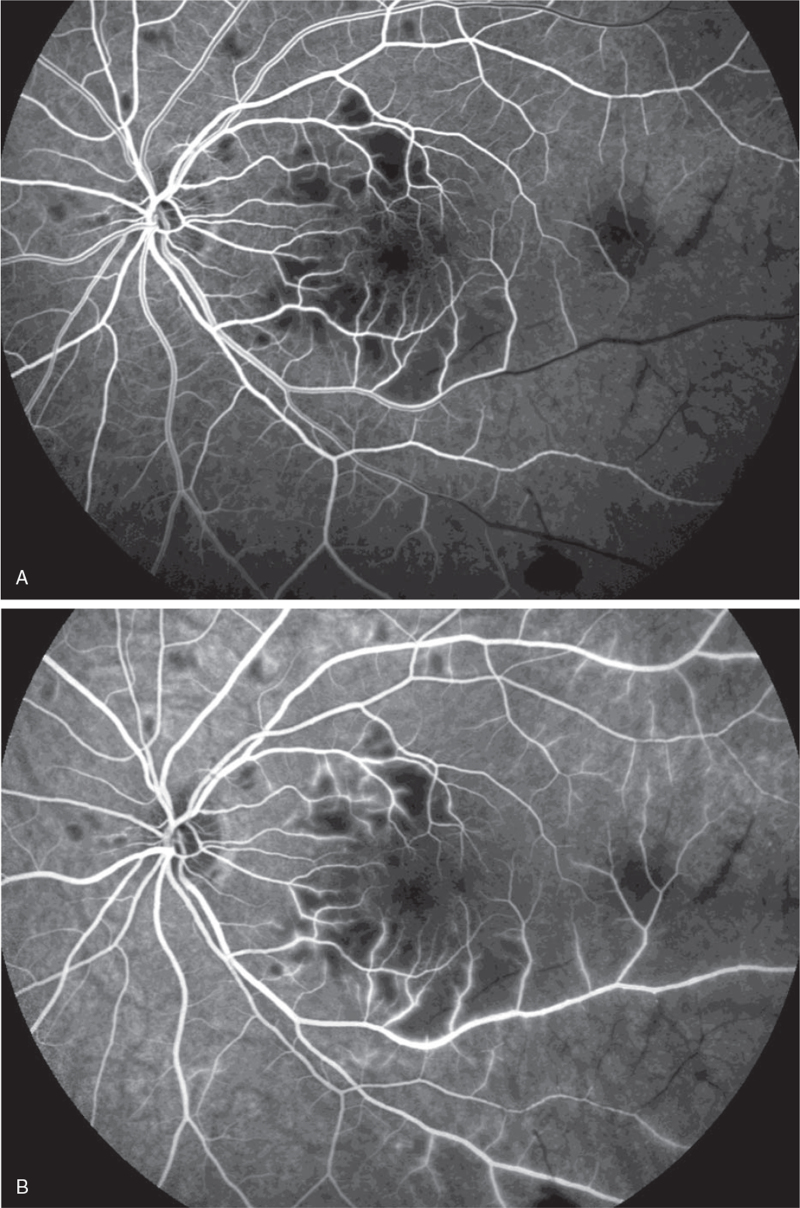
FFA image of the left eye. (A) is the early image of the left eye angiography, the inferior temporal and supratemporal retinal arteries were obviously thinner. The arteries are segmentally non-perfusion; (B) is during the mid-stage angiography, the filling front was still visible in the middle-distal part of the inferior temporal artery, and the large subtemporal small blood vessels were not visible; (C) is the late image of the angiography, with leakage of the venous wall accompanied by staining. During the radiography, there was no nonfluorescent filling in the multiple ischemic areas scattered around the macula.

Ocular examination revealed the best corrected visual acuity (BCVA) of the left eye was 20/50; the BCVA of the right eye was 20/20. Intraocular pressure and anterior segment examination was normal. On fundus examination (Figs. 1 and 2), in left eye there were several cotton-wool spots near the nasal and upper branch artery. The macular was edema, with multiple gray-white ischemic area around. Arteriolar attenuation was present near macula, middle-distal part of which was white thread-like occlusion. The branch retinal veins were slightly tortuous and dilated. In mid-peripheral and peripheral retina, blot hemorrhages and white-line like occluded arterioles can be clearly seen. This fundus image was suggestive of a vaso-occlusive retinopathy in left eye, for comparison, there is no abnormality in the right eye. Cystoid macular edema of the left eye was confirmed by frequency-domain optical coherence tomography (OCT) (Fig. 3). Fundus fluorescein angiography (FFA) (Fig. 4) showed multiple ischemic areas in the area around the macula, arteriolar attenuation, and nonperfusion arteries in temporal and superior temporal. Leakage could be seen in the branch veins at the late stage of the angiography. The fundus of the right eye, OCT and FFA were normal.

**Figure 5:**
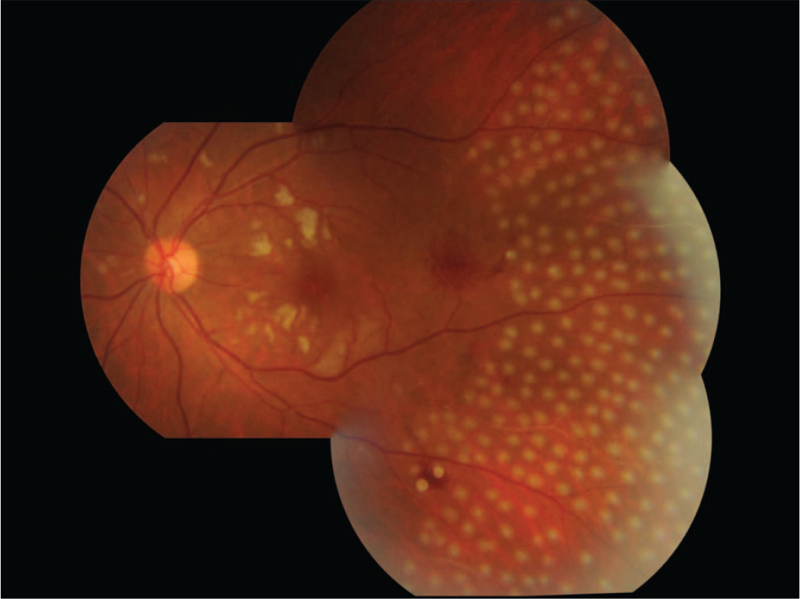
Color fundus image of the left eye after retinal laser photocoagulation. The occluded areas of temporal and inferior temporal branches were laser sealed.

Branch retinal artery occlusion (BRAO) was initially proposed. The patient received moderate flow oxygen inhalation and treatments on left eye including ocular massage, anisodamine hydrochloride posteye injection, and 2% brimonidine tartrate eye drops. Laser local retinal photocoagulation in the left eye was performed to seal the occluded area of the temporal and subtemporal branch vessels (Fig. 5). 2 days after all the treatments above, the patient still complained of blurred vision in her left eye. At this time, the BCVA of her left eye was 20/40 and the macula was still obviously edema.

**Figure 6:**
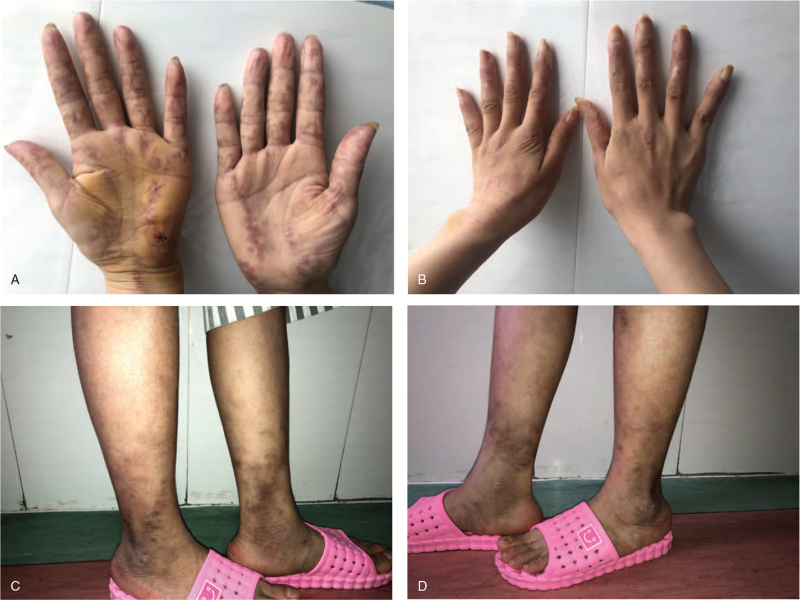
Physical examination. The palms, knuckles, interphalangeal joints of both hands, the distal ends of the lower limbs, and the ankles were scattered with dark red atrophic rashes of various sizes, irregular shapes, unclear boundaries.

A systemic physical examination revealed significant signs: scattered dark red atrophic rashes on palms, finger joints, interphalangeal joints, bilateral lower limbs, and ankles (Fig. 6). She mentioned that these rashes appeared a year ago. She recalled a short-term hair loss 6 months ago, and bilateral knee and ankle pain 3 months ago, for which she didn’t see a doctor and didn’t get treatment. Further laboratory tests provided very meaningful results: red blood cell count was 4.95 ∗10^12^/L, hemoglobin measurement was 103 g/L, average red blood cell volume was 69.9 fl, average Red blood cell hemoglobin was 20.8 pg indicating that the patient has small cell hypochromic anemia; thrombocytopenia (platelet count was 166 ∗10^9^/L); erythrocyte sedimentation rate was elevated to 33 mm/h; multiple vasculitis and autoantibodies were positive including anti-SmD1 (++), anti-U1-snRNP (++), anti-P0 (rRNP) (++), anti-double-stranded DNA (++), anti-histone (+), SSA/Ro60KD (+). Based on the above medical history, physical signs, and laboratory test results, the patient meets the diagnostic criteria for SLE.

**Figure 7:**
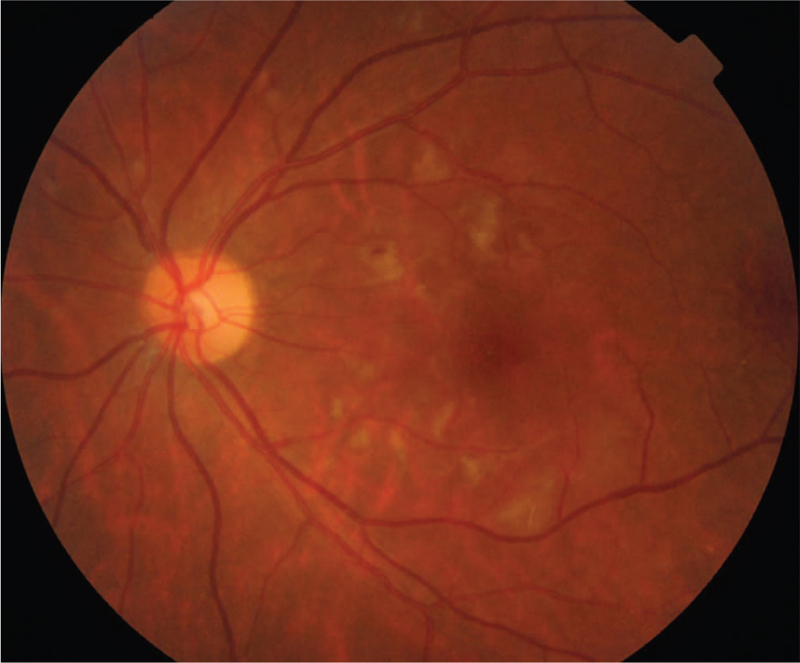
Fundus image of the left eye 10 days after discharge from the hospital. The gray-white edema areas of the retina around the macula were significantly smaller than before. Cotton-wool spots were reduced.

**Figure 8:**
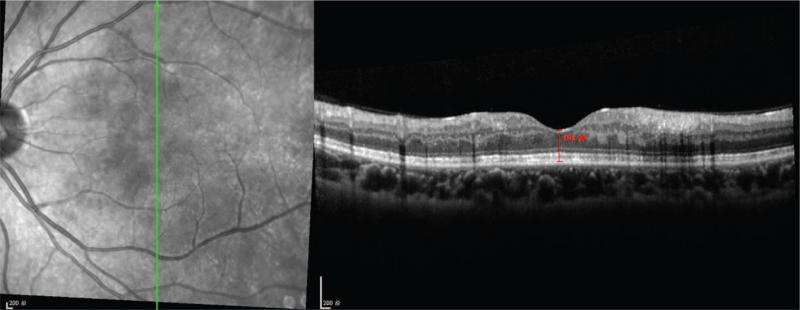
SD-OCT image of the left eye 10 days after discharge from the hospital. Macular edema was significantly alleviated compared to before.

**Figure 9:**
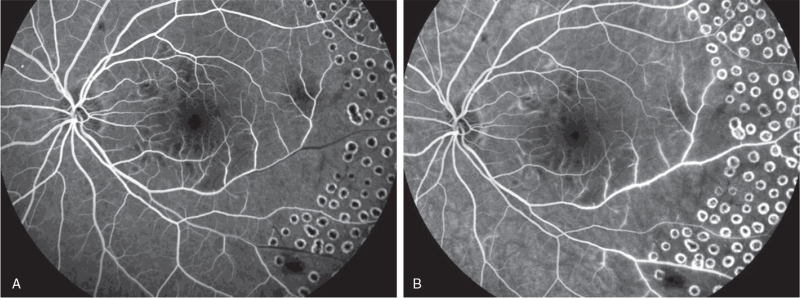
FFA image of the left eye 10 days after discharge from the hospital. The nonfluorescent perfusion area around the macula was reduced than before. Some small arteries were still perfused slowly or without perfusion.

The patient was transferred to the department of Renal Rheumatology and Immunology, where she received treatment including intravenous infusion of Methylprednisolone 120 mg qd for 5 days, oral Methylprednisolone 32 mg qd and oral Mycophenolate mofetil 750 mg bid for 4 days. We followed up the patient 10 days after she was discharged, when she was still taking methylprednisolone 32 mg qd. The cotton-wool spots were reduced than before (Fig. 7). The left eye BCVA was surprisingly improved to 20/20. The Spectral-domain optical coherence tomography revealed that macular edema had almost disappeared (Fig. 8). FFA showed that blood perfusion was partially restored in the previously nonperfused area of the retina (Fig. 9). In a year of follow-up since then, the patient did not experience any recurrence of vision loss. No adverse or unexpected events occurred.

## 3 Discussion

SLE is an autoimmune inflammatory disease that affects multiple organs. The eye can be involved due to the thrombotic and inflammatory phenomena, resulting in keratoconjunctivitis sicca, scleritis, uveitis and ischemic optic neuropathy.^[10]^ BRAO as the sole presentation of retinopathy in SLE is extremely rare. Bilateral BRAO has been reported twice in SLE.^[11,12]^ Only one case series recorded cases of unilateral BRAO with nonischemic central retinal vein occlusion.^[11]^ As far as we know, this is the first case report of unilateral BRAO as primary manifestation of retinopathy in SLE. The patient presented mainly the symptoms of BRAO, such as acute visual impairment, occlusion of branch retinal artery, and macular gray-white edema, which can easily cause misdiagnosis. It is worth noting that in primary BRAO, multiple branch retinal arteries are rarely involved in fundus at the same time, and retinal hemorrhage is uncommon. However, eye examinations sometimes may not be sufficient to reveal the correct diagnosis. In our case, the patient's history of skin rash, hair loss, and joint pain provided the key evidences for SLE diagnosis. Other probable cause for the occlusion of small arteries were examined, such as Giant Cell Arteritis regarding Erythrocyte sedimentation rate was elevated (33 mm/h). Giant Cell Arteritis was excluded due to the patient being in the atypical age group, and the absence of typical features such as headache and symptoms of cranial artery insufficiency. Another possible cause was sickle cell retinopathy. Laboratory examination indicated that the patient was anemia. After consultation with a hematologist, with a negative hemolysis test, we ruled out sickle cell retinopathy.

The common finding of SLE retinopathy is described as kind of Purtscher-like retinopathy (Purtscher retinopathy refers to a special retinopathy that occurs after severe head trauma). The fundus could be manifested as cotton-wool spots around the optic disc, optic disc oedema, yellow-white patches of the retina, and hemorrhage. The pathological basis of SLE retinopathy has often been alluded in literatures as an immune complex mediated ’vasculitis’.^[6]^ It is classically believed that inflammatory immune complexes cause fibrinoid degeneration, necrosis, and thrombosis in the connective tissues of the retina and small blood vessel walls, blocking the small blood vessels of the retina and leading to hemorrhage and ischemia. However, the exact nature of these occlusive lesions is unclear. In cases of central nervous system lupus, pathological studies found non-inflammatory vascular occlusions rather than true arteries,^[13]^ which is consistent with our case. The evidence of active retina vasculitis such as extensive and diffused exudation and bleeding was not observed. The patient presented with multiple segmental BRAO with diffuse capillary nonperfusion and small arterial or arteriolar occlusion in the retina. It is classically believed that such microangiopathy is characterized by microthrombosis.^[11,14]^

It has been supposed that SLE patients with increased serum levels of anti-phospholipid antibody (APLA) have a higher propensity for vascular occlusions, since thrombosis during SLE is strongly associated with the APLAs.^[15]^ In a previous case of vaso-occlusive retinopathy in SLE, which involved bilateral eyes, antiphospholipid antibodies presented positive.^[14]^ The author attributed the phathogenic mechanism to the role of thrombosis associated with the antiphoslipid syndrome. However, in our case, the APLA screening for lupus anticoagulant and anti-cardiolipin antibody did not show abnormal. But in our patient, vaso-occlusive retinopathy developed despite normal APLA. For such presentation suggesting that APLAs are not the only pathogenic factor for the thrombosis during SLE. The patient's anti-dsDNA antibody and anti-sm antibody were both significant positive. A recent research revealed a great important role of anti-dsDNA Abs in promoting thrombosis through platelet dysfunction.^[16]^ The effect anti-dsDNA Abs on platelets manifested as direct activation via the FcγRIIA receptor and structural alterations.^[16]^ The activated platelets promote a pro-thrombotic state and impair contraction of intravital clots,^[17]^ contribute to high incidence of arterial and venous thrombosis in SLE patients. The pathogenesis of simultaneous BRAO/Central retinal artery occlusion in SLE is proposed to be associated with hypercoagulable state.^[11]^ In our case, the coagulation test of this patient did not reveal any abnormal indicator. While laboratory test found thrombocytopenia, with platelet count 166 ∗10^9^/L (average platelets are 266∗10^9^/L). One possible explanation of low platelets is that hyper-production of anti-ds-DNA antibody led to systemic platelet activation, making platelets highly adhesive to the vessel wall and to each other, which not only caused platelet consumption but also provided a strong predisposition to thrombosis.

In the course of SLE with posterior segment involvement, the patients are very likely to have severe visual impairment. Vision loss in 80% of case and neovascularization in 40% of case have been reported in SLE retinopathy patients.^[14]^ However, until now there is no clear consensus on medical treatment of retinal vaso-occlusion in SLE. The close association between SLE retinopathy and systemic inflammatory activities emphasis the great importance of systemic immunotherapy treatment. It has been suggested that systematic treatment should be taken once SLE retinopathy being found. In a previous reported case of BRAO in SLE patient, 3 dose of intravitreal ranibizumb injection were applied, together with anticoagulant, steroid and immunosuppressive therapy under the guidance of a rheumatologist. However, postintervention BCVA did not improve significantly. In our case, the patient received a combination of steroid immunotherapy and immunosuppressive therapy, without any intravitreal anti-VEGF therapy, which achieved an inspiring result. The erythema on the extremities was reduced, and the macular edema was almost eliminated. BCVA of the patient's left eye was improved to 20/20, indicating that immunotherapy is of great significance to patients with SLE retinopathy. Besides, since FFA showed no perfusion of the retina around the vascular occlusion area, retinal laser photocoagulation was conducted to prevent retinal neovascularization. When the patient's steroid dose was slowly reduced until the drug was discontinued, she had never experienced vision loss or recurrence of macular edema in the following one-year follow-up. The patient was very satisfied with the treatment.

There are 2 major limitations in this study that could be addressed. First, the study focused on the ocular symptoms of patients, and the immune index changes in the laboratory tests of the patient were not tracked, which is also an indicator of the treatment effect. Second, retinal artery occlusion in this case was moderate and the central retinal artery was not blocked, thus also contributing to the good treatment outcome. However, the treatment of this case may not be applicable to more severe cases.

This case presented an atypical unilateral SLE retinophathy, with simultaneous BRAO as the primary manifestation. This case emphasized the importance of systemic immunotherapy for SLE, even if the patient's main symptoms are in the retina.

## Acknowledgments

We thank Li Yanhao, the examiner of the Ophthalmology Department of Foshan Second People's Hospital, for providing valuable imaging data.

## Author contributions

**Conceptualization:** Linglin Zhang, Yan Lu.

**Data curation:** Linglin Zhang, Chaoqiang Guan.

**Investigation:** Linglin Zhang, Yan Lu.

**Methodology:** Linglin Zhang, Chaoqiang Guan, Zuke Ye.

**Resources:** Linglin Zhang.

**Supervision:** Zuke Ye, Yan Lu.

**Writing – original draft:** Linglin Zhang.

**Writing – review & editing:** Chaoqiang Guan, Yan Lu.
